# Are Preoperative Kattan and Stephenson Nomograms Predicting Biochemical Recurrence after Radical Prostatectomy Applicable in the Chinese Population?

**DOI:** 10.1155/2013/506062

**Published:** 2013-03-05

**Authors:** Victor H. W. Yeung, Yi Chiu, Sylvia S. Y. Yu, W. H. Au, Steve W. H. Chan

**Affiliations:** Division of Urology, Department of Surgery, Queen Elizabeth Hospital, Hong Kong, China

## Abstract

*Purpose*. Kattan and Stephenson nomograms are based on the outcomes of patients with prostate cancer recruited in the USA, but their applicability to Chinese patients is yet to be validated. We aim at studying the predictive accuracy of these nomograms in the Chinese population. *Patients and Methods*. A total of 408 patients who underwent laparoscopic or open radical resection of prostate from 1995 to 2009 were recruited. The preoperative clinical parameters of these patients were collected, and they were followed up regularly with PSA monitored. Biochemical recurrence was defined as two or more consecutive PSA levels >0.4 ng/mL after radical resection of prostate or secondary cancer treatment. *Results*. The overall observed 5-year and 10-year biochemical recurrence-free survival rates were 68.3% and 59.8%, which was similar to the predicted values by the Kattan and Stephenson nomograms, respectively. The results of our study achieved a good concordance with both nomograms (Kattan: 5-years, 0.64; Stephenson: 5-years, 0.62, 10-years, 0.71). *Conclusions*. The incidence of prostate cancer in Hong Kong is increasing together with the patients' awareness of this disease. Despite the fact that Kattan nomograms were derived from the western population, it has been validated in our study to be useful in Chinese patients as well.

## 1. Introduction

Patients' awareness on prostate cancer is increasing together with its incidence in Hong Kong [1]. According to the latest publication by the Hong Kong Cancer Registry in 2008, prostate cancer is the third most common and the fifth leading cause of cancer deaths in Hong Kong males [1]. An objective tool, such as the Kattan and Stephenson nomograms [2, 3], to estimate the expected outcome would be essential for surgeons in decision making and in counseling the patients on prognosis after treatment. Kattan nomograms were first developed in 1998 [2] and later enhanced by Stephenson et al. in 2006 [3], based on the outcome results of patients with prostate cancer recruited in the USA. It has been validated by patient populations from the United States, Australia, and Europe [4–8], but its applicability to Chinese patients is yet to be validated. We aim at studying the predictive accuracy of Kattan nomograms in the Chinese population based on our center's results.

## 2. Patient and Methods

From 1995 to 2009, 408 patients who underwent laparoscopic or open radical prostatectomy for prostate carcinoma were recruited. The clinical stage of prostate cancer (according to 2009 TNM classification system), the pre-op PSA level, the number of positive and negative cores by transrectal ultrasound guided prostate biopsy, and Gleason Scores of the specimens were collected for analysis. All patients were followed up regularly with PSA monitored, and biochemical recurrence was defined as two or more consecutive PSA level greater than 0.4 ng/mL after radical prostatectomy or secondary cancer treatment [2]. 82 patients who had missing data (such as PSA and Biopsy results) or defaulted followup were excluded from the study. The observed biochemical recurrence rates, calculated by the Kaplan-Meier analysis, were compared with the values predicted by the Kattan nomograms. In order to facilitate the calculation of the concordance index (Range: 0.5 to 1) [9–12], the population was divided into 5 groups (based on similar Kattan scores) with almost equal number of patients in each group. Concordance index can be defined as the proportion of randomly paired patients for whom the patient with the greater probability of recurrence also had earlier disease recurrence [2, 4, 6, 9–12]. SPSS version 19 was used for all statistical analyses.

## 3. Results

**Figure 1:**
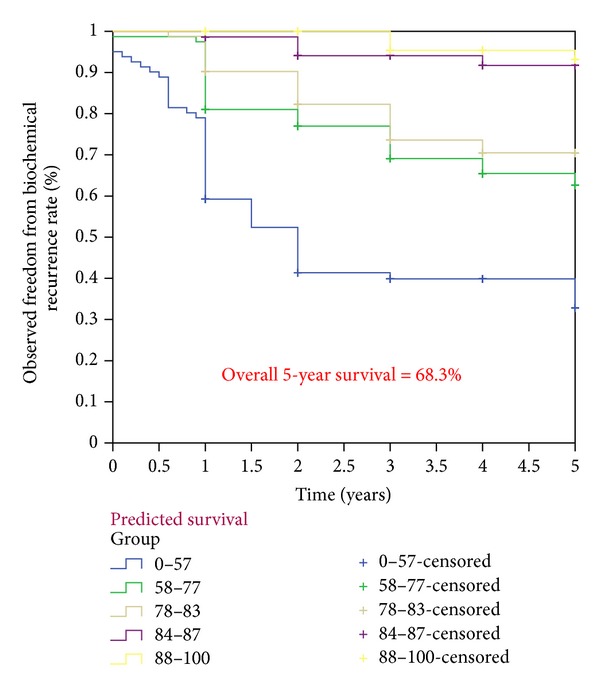
Observed freedom from biochemical recurrence rates according to the 1998 Kattan nomogram.

**Figure 2:**
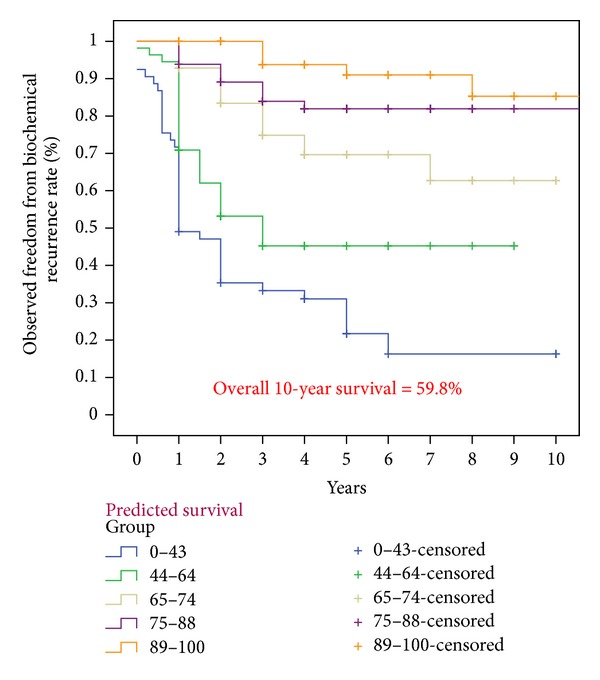
Observed freedom from biochemical recurrence rates according to the 2006 Stephenson nomogram.

**Figure 3:**
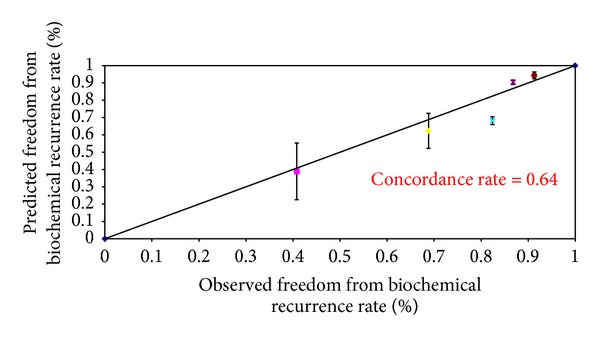
Observed versus predicted 5-year freedom from biochemical recurrence rates according to the 1998 Kattan nomogram.

**Figure 4:**
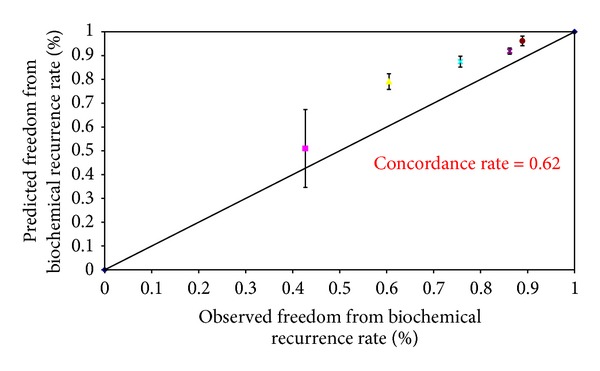
Observed versus predicted 5-year freedom from biochemical recurrence rates according to the 2006 Stephenson nomogram.

**Figure 5:**
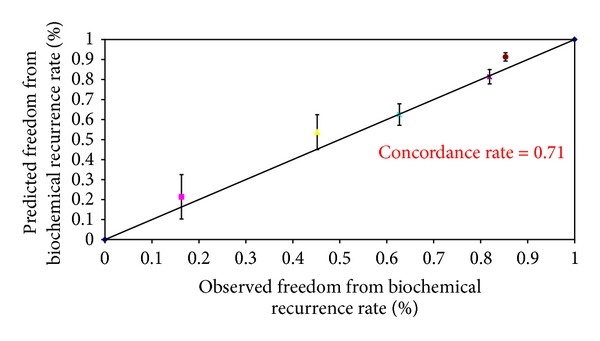
Observed versus predicted 10-year freedom from biochemical recurrence rates according to the 2006 Stephenson nomogram.

The baseline demographics of the patients in our cohort were shown to have higher PSA values and Gleason scores as compared to the Kattan cohorts (Table 1). Thus, the overall observed 5-year (68.3%) and 10-year (59.8%) biochemical recurrence free survival rates were slightly lower than those of Kattan's cohort (Figures 1 and 2). However, these two observed values were similar to the predicted values derived from the 1998 Kattan and 2006 Stephenson nomograms. The plots comparing the observed and predicted 5-year and 10-year biochemical recurrence rates demonstrated that our study results achieved a good concordance with both Kattan nomograms (Figures 3, 4, and 5). For the 1998 version, the 5-year concordance rate was 0.64, whereas for the 2006 version, the 5-year and 10-year values were 0.62 and 0.71, respectively. 

## 4. Discussion

The incidence of prostate cancer is increasing in Hong Kong with 1369 new cases diagnosed in 2008 [1]. An objective tool to estimate the expected outcome would be essential for surgeons in decision making and counselling the patients on prognosis after surgical treatment. There are multiple nomograms generated for the calculation of post-prostatectomy outcomes [13–18]. Kattan nomogram is the one of the most accepted guidelines in determining the biochemical recurrence-free survival rate after prostatectomy. It was generated based on a cohort of patients with prostate cancer recruited in the USA, and had been validated in various studies [4–8]. However, there is a possibility that the Kattan nomograms might not be applicable to Chinese population, because of the different risks of prostate cancer between Asian and Western populations [19–25]. Thus, we perform the validation of Kattan nomograms in Chinese patients with our data collected in Hong Kong.

We have inferior patient background demographics as compared to the Kattan cohort, and thus, our 5-year and 10-year biochemical recurrence rates were higher. However, our data demonstrated a good concordance index with the expected results. The overall observed 5-year and 10-year biochemical recurrence free survival rates were similar to the predicted values by the 1998 Kattan and 2006 Stephenson cohorts respectively. The concordance indexes of our study (5 year, 1998 version: 0.64, 5-year and 10-year, 2006 version: 0.62 and 0.71) were similar to those of Kattan's external validation cohort [2] (5-year: 0.64) as well as Stephenson's cohort [3] (5-year: 0.6, 10-years: 0.71). Other studies (Eskicorapci et al. [6]—5-year, 1998 version: 0.698, 10-year, 2006 version: 0.705 and Jr. et al. [5]—5-year: 0.74) in Turkish and African men also showed comparable concordance indexes as to our study. This suggested that the Kattan nomograms could be applied to different populations over the world, including the Chinese population as supported by our data. Despite the fact that the Kattan nomograms showed a good concordance with our cohort results, the 2006 version slightly overestimated the 5-year biochemical recurrence free survival rates.

There were several limitations in our study. First, our data was based on a single institution, though the operations were performed by various surgeons. Second, our cohort population size was relatively small, and a larger sample size may demonstrate a better concordance with the Kattan nomograms.

## 5. Conclusion

Incidence of prostate cancer in Hong Kong is increasing together with patients' awareness on this disease. Despite the fact that Kattan nomograms were derived from the western population, it has been validated in our study to be useful in Chinese patients as well. The 1998 Kattan nomogram can slightly better predict the outcome of the 5-year biochemical recurrence free survival than the 2006 version. The 2006 version achieves a satisfactory concordance with the 10-year outcome of our cohort. In view of the promising results, Kattan nomograms have been implemented into our clinical practice in managing patients with prostate cancer.

## Figures and Tables

**Table 1:** Background demographics of patients with prostate cancer undergoing prostatectomy.

Variables	Kattan 1998	Stephenson 2006	Our study
PSA	*N* = 983	*N* = 1978	*N* = 326
<4	217 (22.1%)	Median PSA = 6.1 (Range: 4.4–9.0)	28 (8.6%)
4.1–10	472 (48%)		136 (41.7%)
10.1–20	187 (19%)		102 (31.3%)
>20	107 (10.9%)		60 (18.4%)
Clinical stage			
T1a/b	83 (8.4%)	0 (0%)	33 (10.1%)
T1c	148 (15.1%)	803 (40.6%)	224 (68.7%)
T2a	266 (27.1%)	509 (25.7%)	32 (9.8%)
T2b	246 (25.0%)	335 (17.0%)	21 (6.5%)
T2c	182 (18.5%)	244 (12.3%)	10 (3.1%)
T3	58 (5.9%)	88 (4.4%)	6 (1.8%)
Gleason score (GS)			
GS 1-2/1-2	108 (11%)		0 (0.0%)
GS 1-2/3	158 (16.1%)		1 (0.3%)
GS 3/3 and 3/1-2	405 (41.2%)		153 (46.9%)
GS 3/4-5	213 (21.7%)		103 (31.6%)
GS 4-5/1-5	99 (10.1%)		69 (21.2%)
GS 2–6		1348 (68%)	154 (47.2%)
GS 3+4		397 (20%)	98 (30.1%)
GS 4+3		130 (7%)	41 (12.6%)
GS 8–10		104 (5%)	33 (10.1%)
